# Placental mesenchymal dysplasia, a case of intrauterine sudden death of fetus with rupture of cirsoid periumbilical chorionic vessels

**DOI:** 10.1186/1746-1596-6-38

**Published:** 2011-04-24

**Authors:** Takeshi Umazume, Soromon Kataoka, Kyouko Kamamuta, Fumie Tanuma, Akihiko Sumie, Toru Shirogane, Takayuki Kudou, Hitoshi Ikeda

**Affiliations:** 1 Department of Obstetrics and Gynecology, Hakodate Central General Hospital, Honchou 33-2, Hakodate, 040-8585, Japan; 2 Department of Pathology, Hakodate Central General Hospital, Honchou 33-2, Hakodate, 040-8585, Japan

## Abstract

We report a 32-year-old woman (1-gravid, 1-para) with a vesicular lesion in her uterus that was pointed out on ultrasound at 8 weeks' gestation. Amniocentesis at 15 weeks' gestation showed a normal female karyotype, 46XX. As the pregnancy advanced, the mole-like lesion became relatively reduced. Throughout gestation, the maternal human chorionic gonadotropin level was normal, but the serum alpha fetoprotein level rose as her pregnancy progressed. Her fetus did not exhibit any remarkable anomalies. The patient visited our hospital complaining of a diminished feeling of fetal movements at 36 weeks 5 days' gestation, and intrauterine fetal death (IUFD) was confirmed. She delivered a 2336-g female without any definite anomalies. A pathological examination led to a diagnosis of placental mesenchymal dysplasia, and androgenetic/biparental mosaicism in the placenta was identified using p57^kip2 ^immunohistochemical staining. And it also revealed that the rupture of the cirsoid chorionic vessels had led to IUFD.

## Background

Placental mesenchymal dysplasia (PMD) is a rare placental anomaly characterized by placentomegaly and grapelike vesicles resembling molar pregnancy. The incidence of PMD is reportedly 0.02% [1], with a definite preponderance of females [2]. Distinguishing PMD from its mimics, especially molar pregnancy is important for preventing the unnecessary termination of pregnancy. Unlike molar pregnancies, PMD usually features a normal fetus. However, PMD is distinct in that it has a high incidence of fetal growth restriction (FGR) and intrauterine fetal death (IUFD), and is associated with Beckwith-Wiedemann syndrome (BWS), a condition characterized by macrosomia, visceromegaly, macroglossia, and omphalocele [2]. Pathologically, PMD placentas are usually large size, and show edema of stem villi with intact terminal villi and many kinds of vascular anomalies, such as cirsoid chorionic vessels, thrombosis, thickness of vessel wall, vascular stenosis, villous chorangiosis, chorioangioma and fetal thrombotic vasculopathy. Also abnormal umbilical cords, including tortuous, marked twisted cords, excessively long cords, etc. [2,3]. These vascular anomalies may lead to FGR and IUFD. Absence of trophoblastic proliferation in PMD placentas is a histological difference from partial moles [4]. Although the pathogenesis of PMD remains unknown, androgenetic/biparental mosaicism has recently been suggested as the underlying cause of PMD [5]. The phenotypic features of PMD, including the absence of trophoblastic hyperplasia, its association with BWS, and its female preponderance, can all be explained by this novel mechanism. We report a new case of PMD without BWS, but intrauterine sudden death of the fetus without any anomaly contained FGR, at 36 weeks' gestation.

## Case presentation

**Figure 1:**
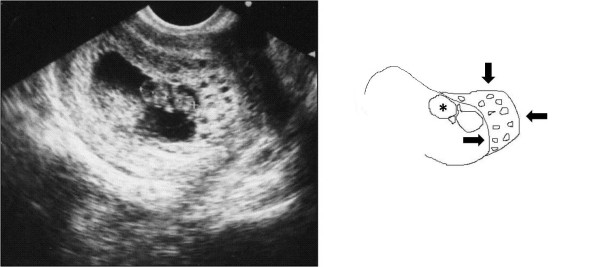
**Transvaginal ultrasound study**. An apparently normal fetus (asterisk) and villi with multiple hypoechoic cysts (arrows) at 8 weeks' gestation. Crown-rump length is 20 millimeters. Each cyst is 2-3 millimeters in diameter.

**Figure 2:**
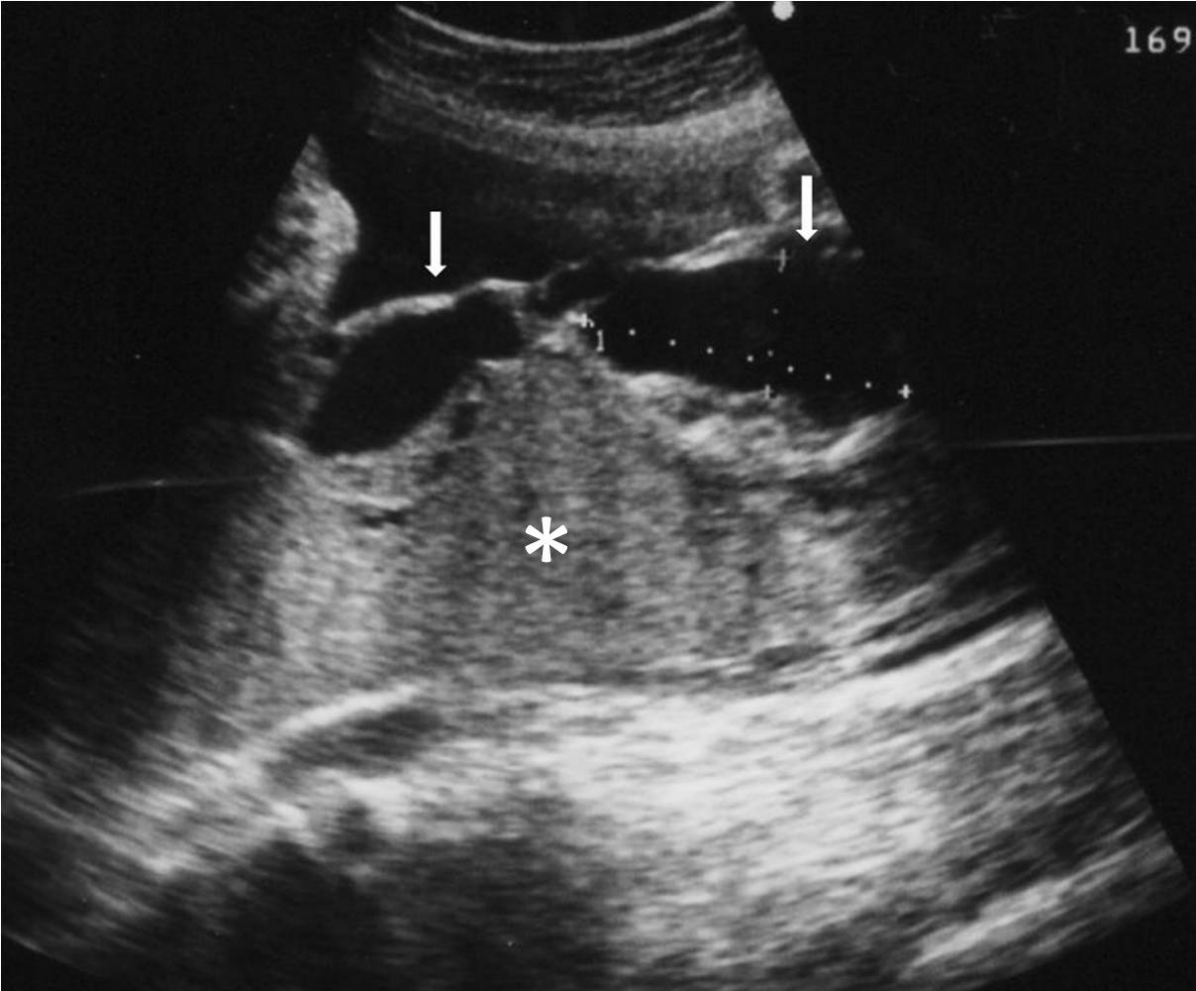
**Transabdominal ultrasound study**. Placental parenchyma (asterisk) and dilated subchorionic vessels (arrows) at 35 weeks' gestation. These vessels are up to 2.5 centimeters in diameter.

**Figure 3:**
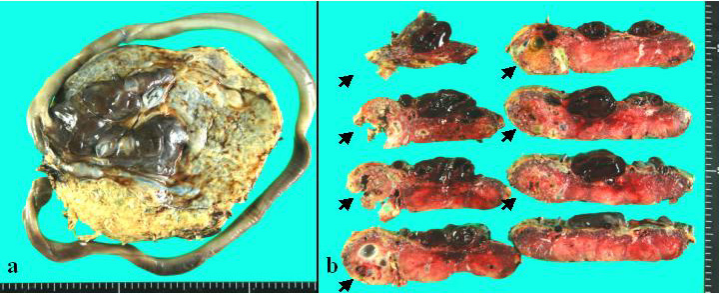
**Fetal surface and cross-section of the placenta**. a) Dilated and tortuous chorionic vessels and an excessively long umbilical cord. b) The formation of a hematoma bordering the dilated chorionic plate vessels and vesicular changes in the parenchyma (arrows).

**Figure 4:**
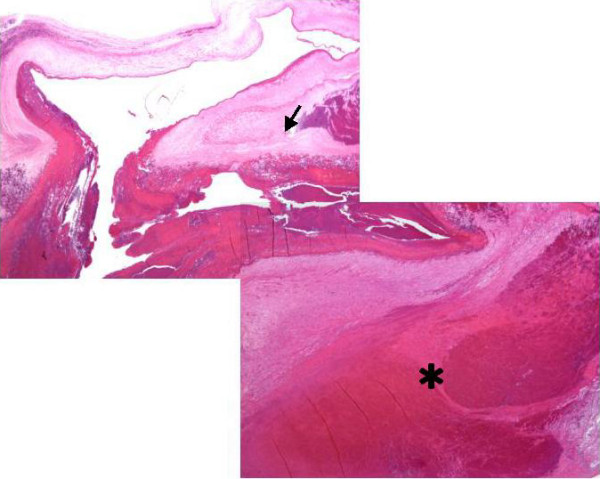
**Merged photographs of a dilated chorionic plate vessel**. The smooth muscle of the dilated chorionic plate vessel has disappeared (arrow), and the vessel wall has ruptured, producing hemorrhage and the formation of a hematoma (asterisk). (hematoxylin-eosin staining (HE), objective ×1). To view the virtual glass slides for this image please see here http://diagnosticpathology.slidepath.com/webViewer.php?snapshotId=1304062157.

**Figure 5:**
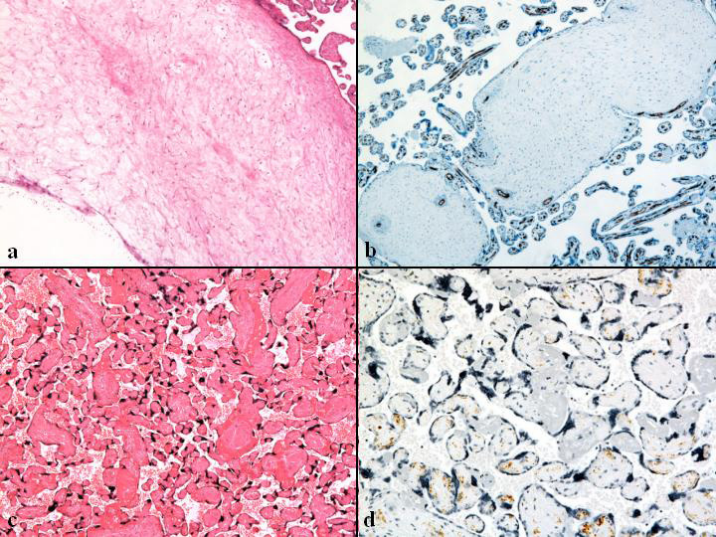
**Histopathology and immunohistochemistry of abnormal villi**. a) Hydropic stem villi and diminished vessels at the affected lesion of the placenta. (HE, objective ×4). The lower left empty corner shows a cystic cavity of the hydropic stem villus. b) Decrease in both number and size of vessels by the CD34 immunohistochemical staining. (objective ×4). c) Avascular terminal villi in a part of the affected area. (HE, objective ×10). d) Scatter and disappearance of immunohistochemical CD34-positive endothelial cells in the avascular terminal villi (objective ×20). To view the virtual glass slide for image 5a please see here http://diagnosticpathology.slidepath.com/webViewer.php?snapshotId=1304062193 and for image 5b here http://diagnosticpathology.slidepath.com/webViewer.php?snapshotId=1304062232. To view the virtual glass slide for image 5c please see here http://diagnosticpathology.slidepath.com/webViewer.php?snapshotId=1304062263 and for image 5d here http://diagnosticpathology.slidepath.com/webViewer.php?snapshotId=1304062298.

A 32-year old woman, gravid 1, para 1, began visiting our hospital at 4 weeks' gestation. An ultrasound performed at 8 weeks' gestation as part of a routine prenatal checkup detected a vesicular lesion in her uterus (Figure 1). The maternal hCG level at 12 weeks' gestation was 100,614 mIU/mL, and this level remained normal throughout gestation. However, the patient's serum alpha-fetoprotein level increased as her pregnancy progressed, reaching 879 ng/mL at 33 weeks' gestation (2.7 multiples of the median; normal medians referenced from previous report [6]). An amniocentesis performed at 15 weeks' gestation showed a normal female karyotype, 46XX. As the pregnancy advanced, the vesicular lesion decreased in size and no fetal abnormalities were detected. At 33 weeks' gestation, however, an ultrasound detected dilated chorionic vessels (Figure 2). The patient visited our hospital complaining of a diminished feeling of fetal movements at 36 weeks 5 days of gestation, and IUFD was confirmed. She delivered a 2336 g female without any definite anomalies at 36 weeks 6 days of gestation. The placenta was 18 centimeters in diameter and weighed 720 g, and a long umbilical cord (about 70 cm) was found. The chorionic vessels were cirsoid, dilated and tortuous (Figure 3a). A cross section of the placenta showed the formation of a hematoma bordering on the dilated chorionic plate vessels and vesicular changes in one-third of the parenchyma (Figure 3b). A histological examination revealed that the cirsoid dilated chorionic vessels were fragile, that a portion of the smooth muscles of the vessel had disappeared, and that the vessel wall was ruptured, causing a hemorrhage with hematoma formation (Figure 4). Hydropic stem villi and diminished vessels were observed in the affected lesion of the placenta. These changes were more clearly shown by immunohistochemical staining with CD34, resulting in the positive staining of vascular endothelium (Figure 5a and 5b). Although almost of the terminal villi were not affected, fetal thrombotic vasculopathy was seen in a part of the affected lesion (Figure 5c and 5d).

**Figure 6:**
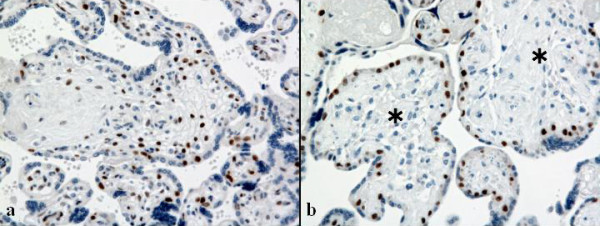
**Androgenetic/biparental mosaicism detected by p57^kip2 ^immunohistochemical staining in the affected lesion**. p57^kip2^-positive stromal cells indicate biparental villi in a, and no p57^kip2^-positive stromal cell in some villi (asterisks) suggests androgenetic villi in b. (objective ×10). To view the virtual glass slide for this image please see here http://diagnosticpathology.slidepath.com/webViewer.php?snapshotId=1304062328.

The p57^kip2 ^gene, which encodes a cyclin-dependent kinase inhibitor, is expressed in the maternal genome, whereas the paternal allele is transcriptionally silent [7]. Immunohistochemical staining of p57^kip2 ^showed that some villi were composed of stromal cells that were negative for p57^kip2 ^only in the affected lesion (Figure 6a and 6b). Therefore, the villi of the affected lesion formed a mosaic of biparental and androgenetic cell populations. These pathological findings led to a diagnosis of PMD.

## Discussion

The main differential diagnoses of PMD are partial hydatidiform mole, dichorionic twins of a normal fetus and complete mole, and confined placental mosaicism. When a prenatal ultrasonographic examination detects a vesicular lesion in the presence of a fetus in uterus, whether a single or twin pregnancy is present should be determined. A twin pregnancy would consist of dichorionic twins of a normal fetus and complete mole. In the present case, ultrasound appeared to reveal a single pregnancy. Unlike partial moles, the majority of PMD are diploid [8]. In 70 - 80% of cases, the partial moles are triploid, often with 2 sets of paternal genes and 1 set of maternal genes as a result of dispermy fertilization [9]. Cases of confined placental mosaicism involving trisomy 16 have been reported as exhibiting cystic villi on ultrasound, but these cases can be diagnosed accurately by karyotyping [10]. In the present case, an amniocentesis performed at 15 weeks' gestation showed a normal female karyotype, 46XX. The characteristic laboratory test for PMD includes an increased level of maternal serum AFP. The increase in the surface transfer area as a result of the increased placental volume and the increased number of vessels within the stem villi is thought to lead to an increased transfer of AFP into the maternal circulation [4]. On the other hand, unlike with moles, the level of hCG in patients with PMD is normal to slightly increased throughout gestation [11]. In this case, although the maternal hCG level was normal throughout gestation, the serum AFP level increased as her pregnancy progressed. These prenatal findings suggested a diagnosis of PMD.

The placenta is usually extremely large for the gestational age and the umbilical cord is relatively long in cases with PMD [2,3]. The gross placental findings for PMD vary with gestational age. To our knowledge, no previous report has detected a vesicular lesion in a patient with PMD prior to 13 weeks' gestation. In the present case, a vesicular lesion was detected at 8 weeks' gestation. Thus, the morphological changes in the villi appear to begin early during gestation. During the third trimester, the chorionic plate vessels in a PMD placenta are aneurysmally dilated and tortuous [11]. However, in cases terminated before 20 weeks of gestation, the chorionic plate vessels are not dilated, and the normal and abnormal areas are not clearly delineated, suggesting that the vascular malformations develop progressively [12]. As the pregnancy advances, tangled congested vessels grossly resembling gray-white or dark-red wormlike structures may be identified within the parenchyma and are often most prominent in the subchorionic plate region near the fetal surface [8]. An ultrasound performed at 33 weeks' gestation seemingly detected this finding (Figure 2).

Although morphologic gross findings of PMD placentas are so often mimicked by features of partial hydatidiform moles, PMD placentas are markedly enlarged and macroscopic vascular anomalies such as cirsoid dilated chorionic vessels with or without thrombi and umbilical cord anomalies like a long umbilical cord that were observed in our case are often co-existence in PMD placentas, but they are not usual in molar pregnancies [2,13]. Microscopically, PMD placentas typically reveal large stem villi with hydropic swelling and cistern formation interspersed with unaffected terminal villi. Similarly, mixture of hydropic villi with cistern formation and unaffected villi are also found in partial hydatidiform moles, however, trophoblastic stromal inclusions and proliferations, which are absent in PMD placentas, are also characteristic of molar pregnancies. Not only macroscopic but also microscopic vascular anomalies such as fetal thrombotic vasculopathy that was found in our case, villous chorangiosis and chorangioma, are also characteristic together hydropic villi in PMD placentas, but not in molar pregnancies [2,3,13]. These morphological characteristics of PMD placenta supported to diagnose our case as PMD. Immunohistochemistry using antibodies against products of paternal imprinting genes such as the antibody against p57^kip2 ^protein is a potential marker that may prove helpful in distinguishing PMD from molar pregnancy. The immunohistochemical detection of androgenetic/biparental mosaicism in stromal cells suggests to a diagnosis of PMD, because this mosaicism is unusual in molar pregnancies [14]. Dichorionic twin placentas with a normal fetus and complete mole may also be needed to pathologically distinguish to PMD. But, vascular anomalies seen in PMD placentas are absent in their placentas and complete moles are purely androgenetic. Spontaneous abortion with hydropic change may have vesicle formation and can also be confused with early PMD, however, the vesicles, if present, are usually small. Also, spontaneous abortion shows degenerative changes [3].

PMD has distinct clinicopathological features. According to Truc et al. [2], 82% of the affected fetuses are female and about 20% of cases with PMD also have BWS. Among the PMD cases without BWS, the FGR and the IUFD rate are 50% and 36%, respectively. IUFD can occur throughout gestation (before 21 weeks, 14.3%; 22~27 weeks, 23.8%; 28~33 weeks, 33.3%; after 34 weeks, 28.6%). The cause of IUFD is currently unclear and may be heterogeneous. Thrombosis of chorionic vessels and umbilical cord anomalies are thought to be likely causes of IUFD in PMD cases, and Truc et al. reported that IUFD may be explained by a potentially chronic hypoxia secondary to obstructive fetal vascular thrombosis and a decrease in maternal-fetal gas exchange as a result of an insufficient amount of normal chorionic villi and the shunting of blood from the exchange surface in chorioangiomas and dysplastic villi [2]. In our case, histological examination revealed that the dilated cirsoid chorionic vessels were fragile and that part of the vessel wall had ruptured, resulting in hemorrhage with hematoma formation. Although fetal thrombotic vasculopathy was found in a part of the affected lesion, this hemorrhage was thought to have led to sudden death of the fetus at the late gestational age (36 weeks 5 days) because no significant chronic hypoxic anomaly of the fetus including FGR was observed.

The underlying cause of PMD is currently unknown. Recently, Kaiser-Rogers et al. proposed androgenetic/biparental mosaicism as the origin of some cases of PMD and suggested that the phenotype of androgenetic mosaicism can presumably range from mild PMD, which may not even be diagnosed, to the typical findings of a complete hydatidiform mole, depending on the extent and distribution of the androgenetic lineage [5]. The authors hypothesized that such mosaicism arose as the result of a failure in the replication of the maternal genome prior to the first cleavage, with normal replication and segregation of the paternal genome, resulting in two types of daughter cells, one with normal biparental genes and the other with only paternal genes. Such failed division would produce a diploid/haploid mosaic embryo, and endoreduplication of the haploid paternal-only daughter cell could then occur to produce the diploid androgenetic lineage, while the female and male haploid complements would merge to form a daughter cell with normal biparental inheritance. The phenotypic features of PMD, including the preponderance of females, the absence of trophoblastic hyperplasia, and the association with BWS, can all be explained by this mechanism. Since an androgenic 46, YY cell line would be nonviable, PMD cases would show the marked female predominance. The abnormal androgenetic cells would be confined to the chorionic mesoderm, membranes, and vessels, whereas the trophoblastic cells would be normal with no evidence of androgenetic cells [5]. This feature would explain the absence of trophoblast overgrowth in PMD in contrast to complete moles, in which androgenetic cells are identified in the trophoblastic cell layer. BWS is a condition of constitutional overgrowth with genetic linkage to chromosome 11p15.5. [15]. Insulin-like growth factor-2 is located in this lesion, and the maternal allele is normally suppressed so that only the paternal gene is expressed. Thus, the loss of 11p15.5. gene imprinting could lead to PMD in some cases.

## Conclusions

PMD should be included in a differential diagnosis of cystic lesions of the placenta by sonography, especially when a phenotypically normal-appearing fetus can be identified. When the prenatal characteristics of PMD, such as an elevated AFP and normal hCG level in the mother, a normal female karyotype revealed by amniocentesis, and dilated subchorionic vessels revealed by ultrasonography during the third trimester, are detected, termination of the pregnancy at an optimal time could be considered, since sudden IUFD including rupture of the cirsoid chorionic vessels might occur at any time throughout gestation.

## Consent

Written informed consent was obtained from the patient for publication of this case report and accompanying images. A copy of the written consent is available for review by the Editor-in Chief of this Journal.

## Competing interests

The authors declare that they have no competing interests.

## Authors' contributions

TU and SK designed the study, performed clinical investigation, and wrote most of the drafts. KK, FT, AS, TS and TK assisted the clinical investigation, and supported to prepare the manuscript. HI performed the histological and immunohistochemical evaluation, and helped rewrite the manuscript. All authors read and approved the final manuscript.
